# Decanoyl-Arg-Val-Lys-Arg-Chloromethylketone: An Antiviral Compound That Acts against Flaviviruses through the Inhibition of Furin-Mediated prM Cleavage

**DOI:** 10.3390/v11111011

**Published:** 2019-10-31

**Authors:** Muhammad Imran, Muhammad Kashif Saleemi, Zheng Chen, Xugang Wang, Dengyuan Zhou, Yunchuan Li, Zikai Zhao, Bohan Zheng, Qiuyan Li, Shengbo Cao, Jing Ye

**Affiliations:** 1 State Key Laboratory of Agricultural Microbiology, Huazhong Agricultural University, Wuhan 430070, Hubei, China; dr.mimran@uaf.edu.pk (M.I.); chenzheng19860227@163.com (Z.C.); wangxugang@webmail.hzau.edu.cn (X.W.); zhoudy6@webmail.hzau.edu.cn (D.Z.); liyunchuan@webmail.hzau.edu.cn (Y.L.); zikaizhao@hotmail.com (Z.Z.); zhengbohan@webmail.hzau.edu.cn (B.Z.); lqylqy6@webmail.hzau.edu.cn (Q.L.); 2 Key Laboratory of Preventive Veterinary Medicine in Hubei Province, College of Veterinary Medicine, Huazhong Agricultural University, Wuhan 430070, Hubei, China; 3 The Cooperative Innovation Center for Sustainable Pig Production, Huazhong Agricultural University, Wuhan 430070, Hubei, China; 4 Department of Pathology, Faculty of Veterinary Science, University of Agriculture, Faisalabad 38040, Pakistan

**Keywords:** flavivirus, Zika virus, Japanese encephalitis virus, furin inhibitor, precursor membrane protein

## Abstract

Flaviviruses, such as Zika virus (ZIKV), Japanese encephalitis virus (JEV), Dengue virus (DENV), and West Nile virus (WNV), are important arthropod-borne pathogens that present an immense global health problem. Their unpredictable disease severity, unusual clinical features, and severe neurological manifestations underscore an urgent need for antiviral interventions. Furin, a host proprotein convertase, is a key contender in processing flavivirus prM protein to M protein, turning the inert virus to an infectious particle. For this reason, the current study was planned to evaluate the antiviral activity of decanoyl-Arg-Val-Lys-Arg-chloromethylketone, a specific furin inhibitor, against flaviviruses, including ZIKV and JEV. Analysis of viral proteins revealed a significant increase in the prM/E index of ZIKV or JEV in dec-RVKR-cmk-treated Vero cells compared to DMSO-treated control cells, indicating dec-RVKR-cmk inhibits prM cleavage. Plaque assay, qRT-PCR, and immunofluorescence assay revealed a strong antiviral activity of dec-RVKR-cmk against ZIKV and JEV in terms of the reduction in virus progeny titer and in viral RNA and protein production in both mammalian cells and mosquito cells. Time-of-drug addition assay revealed that the maximum reduction of virus titer was observed in post-infection treatment. Furthermore, our results showed that dec-RVKR-cmk exerts its inhibitory action on the virus release and next round infectivity but not on viral RNA replication. Taken together, our study highlights an interesting antiviral activity of dec-RVKR-cmk against flaviviruses.

## 1. Introduction

Flaviviruses are arthropod-borne pathogens (arboviruses) that pose a serious threat to global health and cause millions of infections annually. Regardless of extensive research and public health issues, currently there are no specific antiviral treatments in clinical use for flavivirus infections and, despite licensed vaccines, outbreaks still occur, highlighting challenges in executing effective control measures [1]. Some flaviviruses, like Japanese encephalitis virus (JEV), West Nile virus (WNV), and tick-borne encephalitis virus (TBEV) are associated with severe encephalitis; dengue virus (DENV) and yellow fever (YFV), cause hemorrhagic fever; and the newly emerging Zika virus may cause microcephaly in neonates and Guillain–Barré syndrome in adults [2,3]. Flaviviruses are enveloped, icosahedral with positive sense single-stranded RNA viruses that enter host cells by receptor-mediated endocytosis and transport to endosomes, where an acidic condition triggers conformational changes in the envelope (E) protein that prompt virus and host cell membrane fusion [4]. The released genomic RNA is translated to polyprotein precursor of about 3.4 k amino acids in length. This polypeptide gives rise to three structural (core (C), precursor membrane (prM), and envelope (E)) and seven nonstructural (NS) (NS1, NS2A, NS2B, NS3, NS4A, NS4B, and NS5) proteins by host cell signalases and virus-encoded proteases [5]. NS proteins are mainly involved in virus replication and evasion from host immune response while structural proteins are responsible for virus assembly and successful viral entrance into and exit from host cells [6,7]. 

Immature virions assemble in the endoplasmic reticulum (ER). These non-infectious, immature viral particles contain heterodimers of E and prM proteins [8,9]. Maturation of virions occurs after the exocytic pathway in the trans-Golgi network (TGN) through the host cell endoprotease, called furin [10,11]. This calcium-dependent endoprotease cleaves prM to M protein in an acidic environment of the TGN after recognition of the sequence R-X-R/K-R in all flaviviruses. Mature particles then become infectious and are released in the extracellular environment by exocytosis [12]. This furin-mediated cleavage of prM is a critical step for flavivirus assembly and maturation [13]. Inhibition of furin functioning during the viral life cycle may debilitate flavivirus infectivity and pathogenicity.

Decanoyl-Arg-Val-Lys-Arg-chloromethylketone (dec-RVKR-cmk) and hexa-D-arginine (D6R) are small synthetic furin inhibitors that are suitable for clinical purposes. CMK has now been used by many groups as a reference inhibitor to study the effect of furin and related proprotein convertases. It also significantly inhibits viral infection because of its capacity to irreversibly block furin [14,15]. As reported previously, CMK is more effective than D6R in the reduction of HBV replication by inhibiting furin-mediated processing of the hepatitis B e antigen (HBeAg) precursor into mature HBeAg [16]. Dec-RVKR-cmk is a small, synthetic, irreversible, and cell-permeable competitive inhibitor of all proprotein convertases (PC1, PC2, PC4, PACE 4, PC5, PC7, and furin). This peptidyl chloromethylketone is reported to inhibit furin-mediated cleavage and fusion activity of viral glycoproteins, and acts as an antiviral agent against different viruses, including human immunodeficiency virus [17], Chikungunya virus [18], chronic hepatitis B virus, influenza A, Ebola virus infection [16], duck hepatitis B virus [19], and papilloma virus [20]. The structural-activity relationship of dec-RVKR-cmk was studied by Becker and colleagues by replacing the P1-arginine group with 4-amidinobenzylamide and the N-terminal deconyl group with phenyl acetyl group, and they derived a new compound, named phenylacetyl-Arg-Val-Arg-4-amidinobenzylamide, that exhibited more potency to inhibit the cleavage of hemagglutinin of fowl plague virus compared to dec-RVKR-cmk [15]. Smith et al. and Steinmetzer et al. also patented a peptidomimetic furin inhibitor by modifying the C-terminal of dec-RVKR-cmk with decarboxylated arginine mimetics, resulting in highly potent furin inhibitors [21].

There has been skepticism about whether a wide-range inhibitor of furin and other proprotein convertases (PCs) would interfere with normal cellular processes [22,23]. In the case of the PCs family, the concept of redundancy was observed, which constitutes an advantage over the other protease families. According to this concept, it is assumed that the inhibitory effect of a PC inhibitor in normal cells would be minimized by the redundant actions of other co-expressed PCs. Although the mechanism of the redundancy concept is still not well defined [23], this concept has been extensively validated in vitro, and recently has been explored in an animal study. It was observed that liver-specific furin knockout mice showed no obvious adverse effects, thus suggesting that the redundancy effects of other PCs can compensate for the molecular ablation of furin in normal cellular process [24]. Recent developments in medicinal chemistry have explored whether a peptide-based inhibitor has overcome the particular issues associated to their use, bioavailability, and toxicity [23].

The furin-mediated flavivirus maturation encouraged us to evaluate the therapeutic potential of dec-RVKR-cmk against flaviviruses. Our results highlight the efficacy of dec-RVKR-cmk as an interesting anti-flavivirus agent with significant antiviral activities at a non-cytotoxic concentration, suggesting dec-RVKR-cmk as a potential candidate for the treatment of flavivirus.

## 2. Materials and Methods 

### 2.1. Cell Culture and Virus

African green monkey kidney cells (Vero, ATCC-CCL-81) and baby hamster kidney fibroblast cells (BHK-21, ATCC-CCL-10) were purchased from the American Type Culture Collection (ATCC) and cultured and maintained in Dulbecco’s modified Eagle’s medium (DMEM) supplemented by 10% fetal bovine serum (FBS), 100 U/mL penicillin, and 100 mg/mL streptomycin in a 5% CO_2_ incubator at 37 °C. *Aedes albopictus* C6/36 cells (ATCC CRL-1660) were cultured and maintained in Roswell Park Memorial Institute (RPMI) 1640 medium supplemented with 10% fetal bovine serum (FBS), 100 U/mL penicillin, and 100 mg/mL streptomycin in a 5% CO_2_ incubator at 27 °C. The JEV P3 strain (GenBank: U47032.1) was stored in our laboratory and was propagated and titrated on BHK-21 cells. ZIKV-MR-766 strain (GenBank: AY632535.2) was kindly provided by Dr. Xiaowu Pang (College of Dentistry, Howard University, USA) and was propagated and titrated on Vero cells.

### 2.2. Reagents

**Figure 1:**
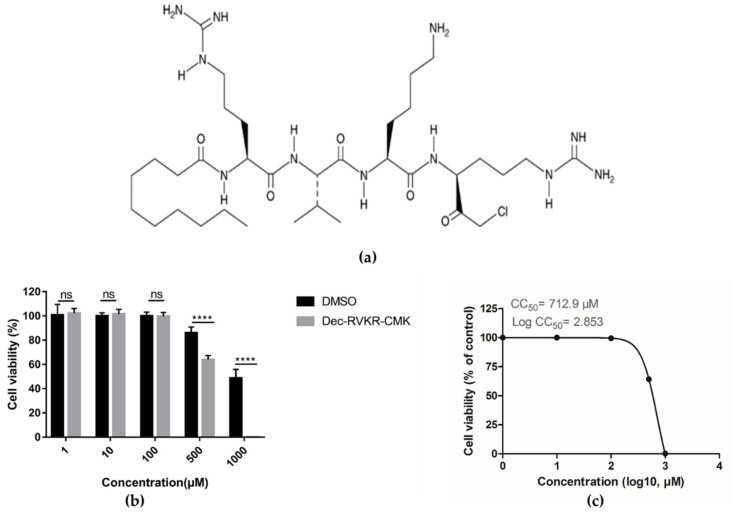
Determination of cytotoxicity of dec-Arg-Val-Lys-Arg-cmk on Vero cells. (**a**) Chemical structure of dec-RVKR-cmk. (**b**) Cytotoxicity of dec-RVKR-cmk on Vero cells determined by CellTiter-GLO One Solution Assay kit (Promega). (**c**) The CC50 value was calculated from GraphPad Prism using non-linear regression analysis. Data are presented as mean ± SEM from three independent experiments.

Dec-RVKR-cmk was purchased from Cayman Chemical (Ann Arbor, Michigan, USA). A stock solution was prepared in dimethyl sulfoxide (DMSO) with a solubility of 33 mg/mL. Further dilutions of this stock solution were made in DMEM prior to performing biological experiments. The structure of dec-RVKR-cmk is shown in Figure 1A. Antibodies against ZIKV prM were purchased from GeneTex (2456 Alton Pkwy Irvine, CA 92606 USA). The monoclonal antibodies against ZIKV (E, NS5) and JEV (prM, E, NS5) were generated in our laboratory. Anti-mouse and anti-rabbit IgG secondary antibodies conjugated with horse reddish peroxidase were purchased from Boster (Wuhan, China).

### 2.3. Cell Viability Assay and Efficacy Study of dec-RVKR-cmk

The cytotoxic concentration 50 (CC50) of dec-RVKR-cmk was determined using the CellTiter-GLO One Solution Assay kit (Promega). This assay was used to detect the viability of cultured cells on the basis of ATP quantification of cells. Briefly, Vero and C6/36 cells were seeded (10,000 cells per well) in a 96-well plate, 24 h before compound treatment. Culture supernatants were replaced with different concentrations of dec-RVKR-cmk or DMSO. Each concentration was tested in triplicate. After 72 h, cells were washed with phosphate-buffered saline (PBS) and an equal volume of (100 µL) CellTiter-GLO reagent was added to each well. For appropriate cell lysis, cells were agitated in a shaker for 2 min and then incubated for 10 min at room temperature. A multimode plate reader was used to quantitate luminescence signals in each condition and then the luminescence value was compared with its corresponding DMSO control. The efficacy of dec-RVKR-cmk against ZIKV (0.2 MOI) and JEV (0.2 MOI) was studied by using different concentrations (1, 10, 50, and 100 µM). The inhibitory concentration 50 (IC50) of dec-RVKR-cmk was determined by counting visible plaques produced by ZIKV or JEV. Both CC50 and IC50 were calculated by non-linear regression model using GraphPad prism7.

### 2.4. Immunofluorescence Assay (IFA)

Vero and C6/36 cells were infected with ZIKV or JEV-P3 at a multiplicity of infection as indicated in the results section for 1 h and the media were replaced with different concentrations of dec-RVKR-cmk or DMSO. Cells were fixed at various time points with ice-cold methanol for 10 min and then washed with PBS. Afterwards, cells were blocked with 10% bovine serum albumin (BSA) in PBS for 30 min at room temperature. Later, cells were incubated with mouse polyclonal anti-NS5 primary antibody of ZIKV or JEV for 1 h at room temperature. After washing with PBS, cells were stained with a second antibody (Alexa Fluor 488) for 30 min at room temperature. Cell nuclei were stained with 6-diamidino-2-phenyl indole (DAPI; Invitrogen). The cells were observed under fluorescence microscope (Zeiss).

### 2.5. Plaque Assay

Viral titers in cell culture supernatants were assessed as described in our previous study [25]. Briefly, virus-infected Vero and C6/36 cells were treated with dec-RVKR-cmk and DMSO. After different time points, as indicated in the results section, virus-containing cell culture supernatants were removed, serially diluted in DMEM, and adsorbed on a Vero (ZIKV) or BHK-21 (JEV) monolayer for 1 h. Afterwards, unbound viral particles were washed and overlaid with 2% carboxymethyl cellulose (CMC). After 5 days of incubation, cells were fixed with 10% formaldehyde for 12 h and then stained with 0.1% crystal violet for 6 h. The visible plaques were counted and viral loads were measured as plaque-forming unit (PFU) per ml of supernatant. All data are expressed as the means of triplicate samples.

### 2.6. Time-of-Drug Addition Assay

ZIKV- (0.2 MOI) and JEV- (0.2 MOI) infected Vero cells were treated with dec-RVKR-cmk under the following conditions: 1 h prior to infection, at the time of infection, or 1, 6, and 12 h post infection (hpi). Regardless of treatment time, cells were infected for 1 h. After 1 h, infectious media were replaced with fresh media and dec-RVKR-cmk added at the above time points. Supernatants were collected at 36 hpi to determine viral titer by plaque assay while cells were used to quantify viral genome copies by qRT-PCR.

### 2.7. Western Blot Analysis

Cells were lysed using radioimmunoprecipitation assay (RIPA) buffer (sigma) containing protease inhibitor (Roche). Samples were mixed with loading buffer and heated at 95 °C for 10 min and then fractionated by SDS-PAGE. Proteins were transferred to a polyvinylidine fluoride membrane (Millipore) using Mini Trans-Blot Cell (Bio-Rad) and blocked with 1% bovine serum albumin. Blots were probed with relevant primary and secondary antibodies and proteins were detected by enhanced chemiluminescent reagent (Thermo Scientific).

### 2.8. RNA Extraction and Quantitative Real-Time PCR

Trizol reagent (Invitrogen) was used to extract intracellular and extracellular RNA and transcribed into cDNA using a cDNA synthesis kit (Toyobo) according to the manufacturer’s instructions. Quantitative real-time PCR was performed using Applied Biosystems 7500 real-time PCR system and TaqMan real-time PCR mix (NovoStart, China). The ZIKV MR-766 E gene or JEV-P3 E gene were used to generate the standard curve for the quantification of viral copy numbers. Each sample was analyzed in triplicate. ZIKV or JEV copy numbers were extrapolated from the generated standard curve using the Applied Biosystems protocol. Primers were as follows: ZIKV, 5’-CCGCTGCCCAACACAAG-3’ (forward) and 5’-CCACTAACGTTCTTTTGCAGACAT-3’ (reverse); ZIKV probe, 5’-AGCCTAACCTTGACAAGCAATCAGACACTCAA-3’; JEV, 5’- TGGTTTCATGACCTCGCTCTC-3’ (forward) and 5’- CCATGAGGAGTTCTCTGTTTCT-3’ (reverse); and JEV probe, 5’-CCTGGACGCCCCCTTCGAGCACAGCGT-3’.

### 2.9. Statistical Analysis

All experiments were performed at least three times with similar conditions. GraphPad Prism, version 7, was used for data analyses of outcomes. Results are presented as the mean ± standard error (SEM). CC50 and IC50 were calculated by non-linear regression. Viral titers are expressed as the medians. Statistical differences were determined by independent t-test or one-way analysis of variance (ANOVA), with Dunnett’s multiple comparison test, and a *p* value < 0.05 was considered as significant.

## 3. Results

### 3.1. Cytotoxicity of dec-RVKR-cmk in Vero Cells

First, we examined the cytotoxicity of dec-RVKR-cmk in Vero cells by using luminescence-based cell viability assay. Viable cells were determined on the basis of ATP quantification of cells, which indicates the presence of metabolically active cells. Different concentrations of dec-RVKR-cmk were used against Vero cells and the results revealed that up to a 100 µM concentration dec-RVKR-cmk exhibited no cytotoxic effect, while 500 and 1000 µM concentrations were significantly toxic to cells (Figure 1b). The CC50 of dec-RVKR-cmk, which is the concentration that results in 50% cell viability, was determined to be 712.9 µM (log CC50 = 2.853) (Figure 1c).

### 3.2. Dec-RVKR-cmk Inhibits prM Cleavage during ZIKV and JEV Infection

**Figure 2:**
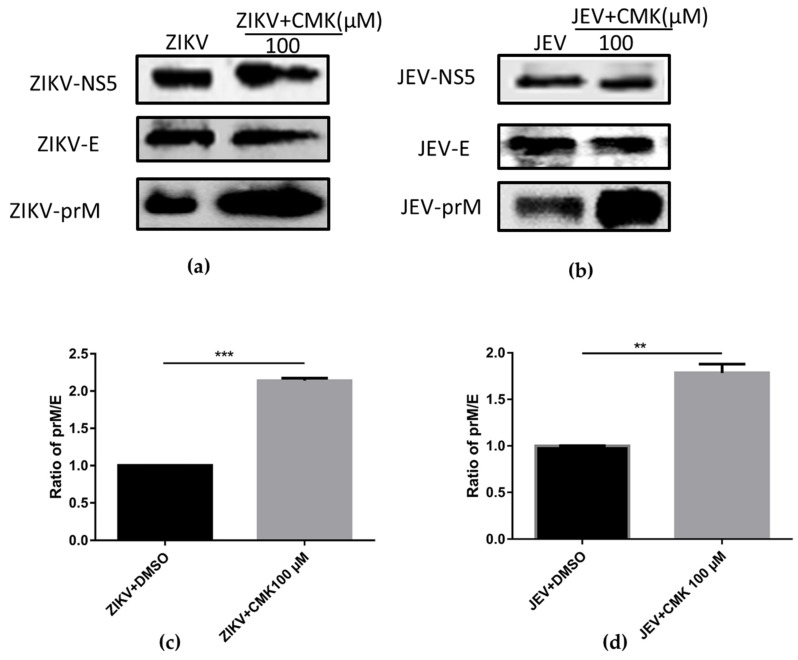
Dec-RVKR-cmk inhibits viral maturation process by preventing prM cleavage. Vero cells were infected with ZIKV-0.2 MOI and JEV-0.2 MOI followed by dec-RVKR-cmk treatment using 100 µM concentration. (**a**) ZIKV and (**b**) JEV viral proteins were analyzed using SDS-PAGE at 36 hpi from the peptidyl CMK-treated and untreated infected cells and then detected by Western blotting (WB) using relevant antibodies. Quantification of E and prM proteins were analyzed by image J software and the ratio of prM/E between the dec-RVKR-cmk-treated and untreated control group was compared for both (**c**) ZIKV and (**d**) JEV.

Flavivirus maturation is associated with the status of the M protein of viral particles, where the prM precursor protein is cleaved in TGN by the host proprotein convertase furin protease. Therefore, cleavage of the prM protein was analyzed by Western blotting (WB) at 36 hpi from Vero cells infected with JEV or ZIKV and treated or untreated with dec-RVKR-cmk. As expected, both dec-RVKR-cmk treated and untreated viral immune complexes showed almost identical bands of E and NS5 proteins, while a prominent thicker band of prM was detected in the dec-RVKR-cmk-treated cells when compared to untreated cells (Figure 2a,b), indicating a larger amount of prM protein accumulated in the treatment group of cells. After that, the relative quantification of detected signals of protein bands E and prM were analyzed by image J software. The proportion of prM was determined by dividing the prM adjusted signal over the E adjusted signal, and then we made a comparison of the prM/E ratio between the ZIKV or JEV treated sample and untreated control. Results revealed a significant increase in the prM/E index in the treated group compared to the untreated control (Figure 2c,d). Taken together, the data suggest that dec-RVKR-cmk exerts its inhibitory action on prM cleavage.

### 3.3. Dec-RVKR-cmk Inhibits ZIKV and JEV Infection at Different Times of Drug Administration

**Figure 3:**
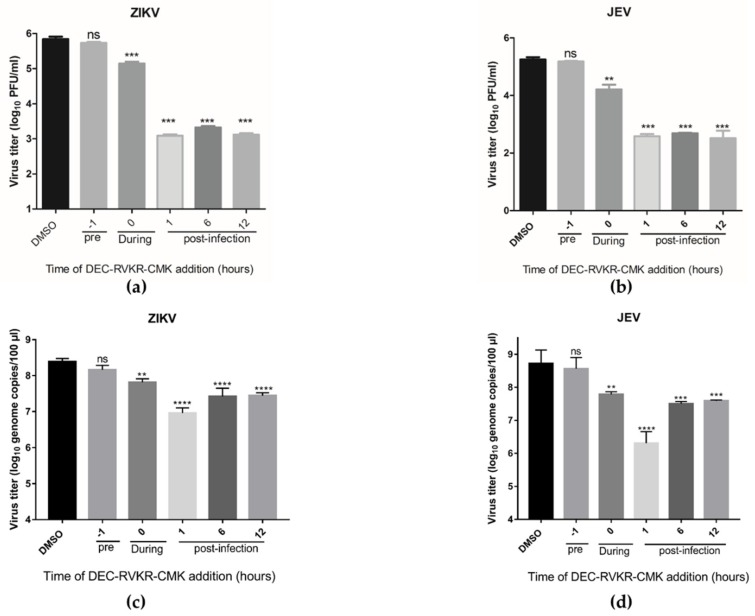
Time-of-addition assay of dec-RVKR-cmk against ZIKV and JEV infection. ZIKV (0.2 MOI) and JEV (0.2 MOI) infected Vero cells were treated with dec-RVKR-cmk (100 µM) at the indicated condition. Time-of-addition study revealed the effect of dec-RVKR-cmk against (**a**,**c**) ZIKV and (**b**,**d**) JEV viral titer production and intracellular genome copies, respectively. Data are presented as the mean ± SEM from three independent experiments.

The ZIKV and JEV life cycle has multiple vulnerable points where suitable therapeutics can potentially be developed. For this purpose, a time-of-addition assay was performed using 100 µM dec-RVKR-cmk added to ZIKV and JEV infected cells 1 h prior to infection, at the time of infection, and 1, 6, and 12 hpi, followed by virus titer determination by plaque assay and quantification of viral genome copies inside the cells by qRT-PCR at 36 hpi. The data indicated that the maximum reduction in both ZIKV and JEV viral titer (Figure 3a,b) and genome copies (Figure 3c,d) were observed when dec-RVKR-cmk was added post infection. Interestingly, the infectious JEV and ZIKV particles released into the media from infected cells and inside the cells were still reduced even when peptidyl cmk was added at the time of infection.

### 3.4. Dec-RVKR-cmk Suppresses ZIKV and JEV Propagation in a Dose-Dependent Manner

**Figure 4:**
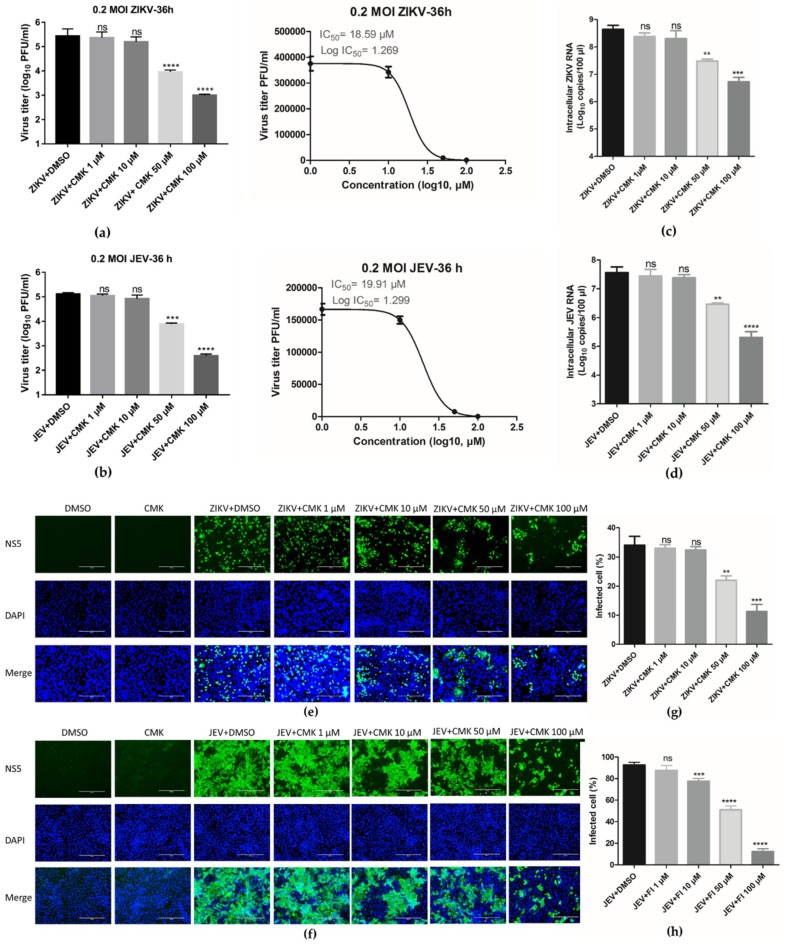
Antiviral assessment of dec-RVKR-cmk against ZIKV and JEV in a dose-dependent manner. Vero cells were infected with ZIKV or JEV at 0.2 MOI followed by dec-RVKR-cmk treatment using the indicated concentration. Right panels (**a**,**b**) indicate virus titer while left panels indicate IC50 of dec-RVKR-cmk against the indicated MOI of ZIKV and JEV. (**c**) ZIKV-0.2 MOI and (**d**) JEV-0.2 MOI infected Vero cells were treated by dec-RVKR-cmk in a dose-dependent manner and then analyzed by qRT-PCR for absolute genome copies using standard curve of in vitro transcribed ZIKV and JEV RNA at 36 hpi. Meanwhile, infected Vero cells with similar condition of RNA analysis were fixed to analyze virus spreading from infected cells to neighboring cells. Immunofluorescence images of (**e**) ZIKV- and (**f**) JEV-infected Vero cells were acquired at 36 hpi, and quantified (**g**) ZIKV and (**h**) JEV immunoreactive positive cells to visualize the inhibition of infection in a dose-dependent manner. Data are presented as the mean ± SEM from three independent experiments.

Next we investigated the antiviral activity of dec-RVKR-cmk against ZIKV and JEV. Vero cells were infected with ZIKV (0.2 MOI) or JEV (0.2 MOI) in the presence of increasing concentrations of dec-RVKR-cmk (1, 10, 50, 100 µM), followed by viral titer determination in supernatant by plaque assay. Dec-RVKR-cmk inhibited ZIKV and JEV in a dose-dependent manner in the viral titer reduction assay. In the case of ZIKV infection, a 1.48 log10 and 2.44 log10 decrease in virus titer was observed with 50 and 100 µM dec-RVKR-cmk treatment, respectively (Figure 4a, left panel). In the case of JEV infection, treatment with 50 µM dec-RVKR-cmk led to a 1.22 log10 decrease in virus titer, while treatment with 100 µM led to a 2.53 log10 decrease in virus titter (Figure 4b, left panel). No significant inhibition of ZIKV and JEV was observed at 1 and 10 µM concentrations of dec-RVKR-cmk. The IC50 of dec-RVKR-cmk, i.e., the concentration at which 50% virus inhibition occurred, was determined to be 18.59 and 19.91 µM against ZIKV and JEV, respectively (Figure 4a,b, right panel).

To obtain a detailed insight into the efficacy of dec-RVKR-cmk against ZIKV and JEV, RT-qPCR was performed. Reduced ZIKV (0.2 MOI) and JEV (0.2 MOI) genome copies were observed in a dose-dependent treatment in Vero cells at 36 hpi. A significant decline in the viral RNA of ZIKV (~2 log10) and JEV (2.2 log10) was observed in the 100 µM treatment of dec-RVKR-cmk. While at the 50 µM treatment, a 1 log10 decrease in ZIKV RNA and a 1.16 log10 decrease in JEV RNA were observed (Figure 4c,d). Treatment with 1 and 10 µM concentrations of dec-RVKR-cmk did not cause significant inhibition of ZIKV and JEV RNA.

Meanwhile, the effect of dec-RVKR-cmk was observed on virus spreading from infected to bystander cells by IFA. Immunofluorescence images of ZIKV- (0.2 MOI) and JEV- (0.2 MOI) infected Vero cells were taken after 36 hpi using different concentrations of dec-RVKR-cmk (Figure 4e,f). The fluorescence signals revealed that a significant ~22.67-fold inhibition of infection was found when ZIKV-infected cells were treated with 100 µM dec-RVKR-cmk and, to a lesser extent, with 50 µM (~12-fold) and with 10 µM (~1.7-fold), as compared to the control (Figure 4g). Similarly, in the case of JEV-infected cells, the counting of immunoreactive cells showed a strong decrease of viral spreading in a dose-dependent manner (Figure 4h). Taken together, the data suggest that dec-RVKR-cmk significantly reduces intracellular and extracellular virus particles along with the inhibition of ZIKV and JEV spreading from infected cells to bystanders in a dose-dependent manner.

### 3.5. Dec-RVKR-cmk Inhibits ZIKV and JEV Propagation at Various Time Points

**Figure 5:**
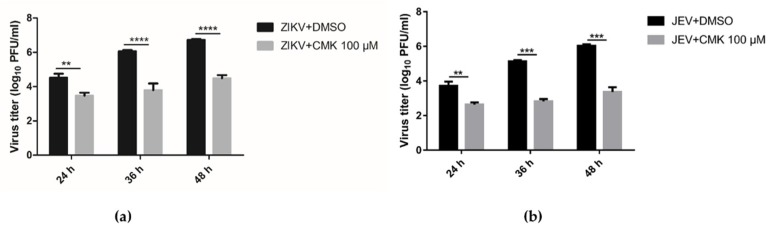

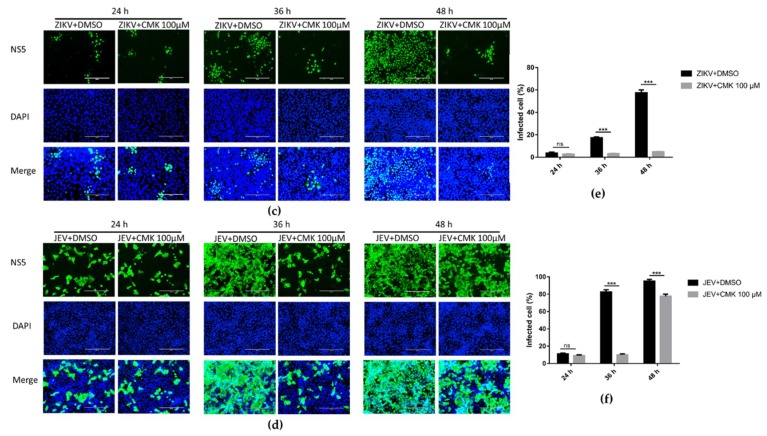
Dec-RVKR-cmk inhibited ZIKV and JEV infection at various time points. Vero cells were infected with ZIKV-0.2 MOI and JEV-0.2 MOI, followed by dec-RVKR-cmk treatment using the 100 µM concentration. Cell supernatant used to determine (**a**) ZIKV and (**b**) JEV viral titer by plaque assay at indicated time points. While immunofluorescence images of (**c**) ZIKV- and (**d**) JEV-infected Vero cells were acquired at different time points in both control (virus + DMSO) and treated (virus + CMK) Vero cells and quantified (**e**) ZIKV and (**f**) JEV immunoreactive positive cells to see the extent of infection. Data are presented as the mean ± SEM from three independent experiments.

We next studied the anti-viral efficacy of dec-RVKR-cmk against both ZIKV (0.2 MOI) and JEV (0.2 MOI) in Vero cells at different time points, i.e., 24, 36, and 48 hpi, using the 100 µM concentration of dec-RVKR-cmk. Cell culture supernatant was used to determine the virus progeny titer by plaque assay. Figure 5a indicates that significant inhibition of ZIKV progeny titer was seen at 24, 36, and 48 hpi (1.6 log10, 2.25 log10, and 2.23 log10, respectively). Similarly, in the case of JEV, significant inhibition was observed at 24, 36, and 48 hpi (1.08 log10, 2.37 log10, and 2.72 log10, respectively) (Figure 5b). For IFA, infected Vero cells were fixed from both the dec-RVKR-cmk-treated and DMSO-treated control groups to see the extent of infection by counting immunoreactive cells. No significant difference in fluorescence signal of ZIKV-NS5 (Figure 5c) and JEV-NS5 (Figure 5d) were observed at 24 hpi between the dec-RVKR-cmk-treated and DMSO-treated control groups. Significant ZIKV (Figure 5c,e) and JEV (Figure 5d,f) inhibition was seen at 36 and 48 hpi, in terms of reduction in the percentage of immunoreactive cells, in the dec-RVKR-cmk-treated group as compared to the control group for ZIKV (14% and 52% inhibition, respectively) and JEV (70% and 23% inhibition, respectively). Thus, the data suggest that the maximum effects of dec-RVKR-cmk were observed at 36 and 48 hpi in terms of reduction of virus progeny titer and inhibition of ZIKV and JEV spreading to neighboring bystander cells.

**Figure 6:**
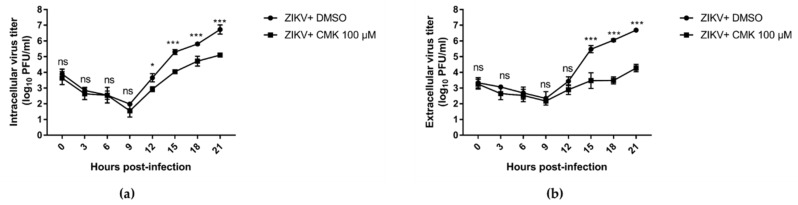
Dec-RVKR-cmk inhibits ZIKV infection in the one-step growth cycle. Vero cells were infected with ZIKV at an MOI of 5 for 1 h followed by dec-RVKR-cmk or DMSO treatment after washing the cells. At the indicated time points after infection, culture medium was collected and cells were subjected to three freeze-thaw cycles to liberate cell-associated viruses. (**a**) Intracellular and (**b**) extracellular virus titer was determined by plaque assay. Data are presented as the mean ± SEM from three independent experiments.

We conducted an experiment to assess virus replication in more detail with the one-step growth cycle. We treated the cells using a 100 µM concentration of dec-RVKR-cmk or DMSO 1 hpi. Vero cells were infected with ZIKV at an MOI of 5 for 1 h and media were replaced with dec-RVKR-cmk or DMSO after washing the cells. The culture medium and cells were collected immediately following infection (0 h) and then every 3 h through 21 hpi. Virus titer was determined in both supernatants and cells by plaque assay on Vero cells. The data indicated that infectious viral particles began to produce between 9 and 12 h post infection in DMSO-treated cells (Figure 6a). Following initial amplification, the subsequent rise was observed at 15, 18, and 21 hpi. Similar findings were observed in the extracellular compartment (Figure 6b). In dec-RVKR-cmk-treated cells, a significant reduction in both intracellular (>1 log_10_ at 15, 18, and 21 hpi) and extracellular (>2 log_10_ at 15, 18, and 21 hpi) virus titers were observed. Taken together, the data suggests that dec-RVKR-cmk inhibits ZIKV and JEV propagation in the one-step growth cycle. Furthermore, the greater reduction in extracellular virus titer than that in intracellular virus titer indicates that dec-RVKR-cmk might inhibit virus release rather than replication.

### 3.6. Dec-RVKR-cmk Inhibits ZIKV Release and Next Round Infectivity

**Figure 7:**
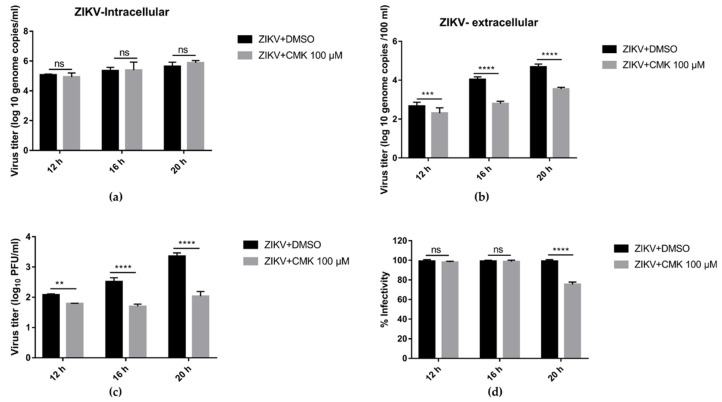
Dec-RVKR-cmk inhibits ZIKV release and next round infectivity. Vero cells were infected with ZIKV (1 MOI) followed by dec-RVKR-cmk treatment using the 100 µM concentration. (**a**) Infected cells were analyzed by qRT-PCR for absolute genome copies using the standard curve of in vitro transcribed ZIKV for the indicated time points. Cell supernatant was used to determine ZIKV viral titer by (**b**) qRT-PCR and also by (**c**) plaque assay. (**d**) The percentage infectivity was calculated by dividing the viral titer determined by plaque assay by the viral titer determined by qRT-PCR in supernatant, for both the control and treated groups, and then the value of the control group was normalized to 100 and compared with the treated group. Data are presented as the mean ± SEM from three independent experiments.

To elaborate on this phenomena, both ZIKV- (1 MOI) infected cells and their supernatants from treatment (dec-RVKR-cmk 100 µM) and control (DMSO) group were subjected to RT-qPCR analysis. Meanwhile, half the volume of supernatant was used to determine viral titer through plaque assay. According to the one-step growth cycle results (Figure 6), 12–21 h may reflect a single round of replication [26]. To clarify at which stage in virus life cycle dec-RVKR-cmk works, both ZIKV- and JEV-infected cells and their supernatants from the treatment (dec-RVKR-cmk 100 µM) and control (DMSO) groups were subjected to RT-qPCR analysis and plaque assay at 12, 16, and 20 hpi. At the intracellular level, there was no difference in genome copies in both the treated and control groups at different time points (Figure 7a). Extracellular viral titer, in terms of genome copies (Figure 7b) and plaque forming unit (Figure 7c), showed a significant difference between the control and treatment groups. Afterwards, we calculated the infectivity of the virus by comparing the viral titer of ZIKV, determined by plaque assay and RT-qPCR in supernatant, for both the control and treated groups. Figure 7d indicates no significant difference in virus infectivity at 12 and 16 hpi, but at 20 hpi a 20% reduction was observed in the released virus. Taken together, these data validate the results, seen in Figure 6, which show that no significant difference in genomic RNA copies was observed in dec-RVKR-cmk-treated and untreated cells at 12, 16 and 20 hpi. Thus, dec-RVKR-cmk cannot inhibit virus replication in the first round of infection but exerts its inhibitory action on virus release and next round infectivity.

### 3.7. Dec-RVKR-cmk Inhibits ZIKV and JEV Infection in Mosquito Cells

**Figure 8:**
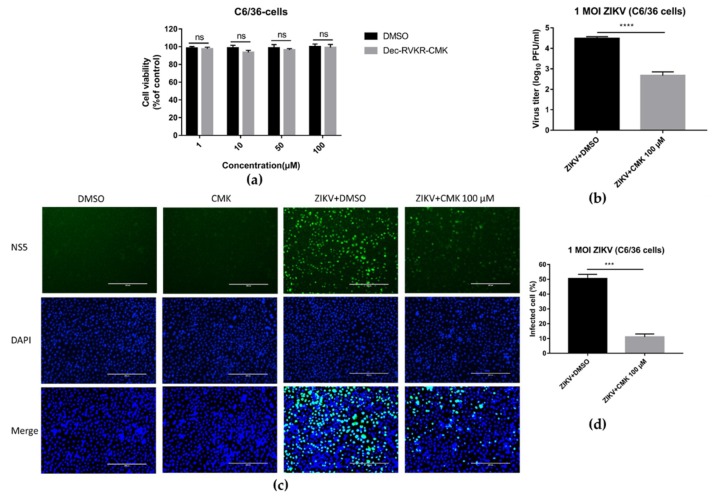
Dec-RVKR-cmk inhibits ZIKV and JEV infection in mosquito cell line C6/36. (**a**) Cytotoxicity of dec-RVKR-cmk in C6/36 cell line was determined by CellTiter-GLO One Solution Assay kit (Promega). (**b**) C6/36 cells were infected with ZIKV (1 MOI) followed by dec-RVKR-cmk treatment using the 100 µM concentration. Cell supernatant was used to determine ZIKV viral titer by plaque assay at 24 hpi. (**c**) Immunofluorescence images of ZIKV-infected c6/36 cells were acquired to quantify (**d**) ZIKV immunoreactive positive cells. Data are presented as the mean ± SEM from three independent experiments.

It is interesting to know whether dec-RVKR-cmk inhibits ZIKV and JEV infection only in mammalian cells or inhibits infection in a mosquito cell line. To address this question, firstly we examined the cytotoxicity of dec-RVKR-cmk to C6/36 cells by using luminescence-based cell viability assay. Likewise, Vero cells treated with dec-RVKR-cmk exhibited no cytotoxicity to C6/36 cell line at the concentrations of 1, 10, 50, and 100 µM (Figure 8a). Afterwards, C6/36 cells were infected with ZIKV (1 MOI) and then treated with 100 µM of dec-RVKR-cmk. Cell supernatant was harvested at 24 hpi for virus titer determination through plaque assay and cells were fixed for immunofluorescence imaging. Figure 8b indicates that a significant inhibition of 2.23 log10 ZIKV progeny titer was observed when compared to the DMSO-treated control. The fluorescence image (Figure 8c) revealed a ~39% reduction in immunonoreactive positive cells in the dec-RVKR-cmk treatment group as compared to the control (Figure 8d). These results suggest that dec-RVKR-cmk could inhibit flavivirus propagation in both mammalian cells and mosquito cells.

## 4. Discussion

Encephalitis, hemorrhagic disease, biphasic fever, jaundice, and flaccid paralysis are typical manifestations of flaviviruses in human beings [2]. Among mosquito-borne flaviviruses, ZIKA and JEV are medically important pathogens. JEV is known to be neurotropic and cause encephalitis, while ZIKV is considered to cause febrile illness. However, in a recent worldwide outbreak, ZIKV has been associated with neurological manifestations, including Guillain–Barré syndrome in adults and fetal microcephaly [27]. Continued rise in flavivirus infections across the world highlights an urgent need for antivirals to combat these challenges.

The objective of this study was to evaluate the antiviral activity of dec-RVKR-cmk against flavivirus. Processing of viral proteins by host cellular protease is a characteristic feature to achieve virus maturation in different viruses of various families. Dec-RVKR-cmk is reported to inhibit cleavage and fusion activity of various glycoproteins mediated by furin and to play a key role in activating several bacteria and viruses, including anthrax, botulinum, influenza A, measles, Ebola, HIV, HBV, and CHIKV [16,28,29,30,31,32,33]. Among flaviviruses, an important step in the production of infectious virions is the processing of prM protein to the anchored membrane M stump and the “pr” peptide that takes place in the TGN by the host proprotein convertase furin protease, prior to release from infected cell [18,34]. Therefore, we assessed furin inhibition activity of dec-RVKR-cmk against ZIKV and JEV. In this respect, our observations confirmed the critical role of furin in flavivirus maturation. We found that peptidyl CMK inhibited ZIKV and JEV more significantly in the later stage of their life cycle by preventing efficient cleavage of prM protein through the host proprotein convertase furin protease resulting in the effective arrest of subsequent viral infection.

The time-of-drug addition assay revealed that dec-RVKR-cmk worked more efficiently post infection but, interestingly, inhibition was also observed when dec-RVKR-cmk added at the time of infection. This antiviral activity of dec-RVKR-cmk was still observed even when the drug was added 12 hpi. Collectively, this suggests that dec-RVKR-cmk is more effective when added post infection. A dose-related inhibition of ZIKV and JEV in Vero cells was observed in terms of extracellular viral progeny titer, intracellular viral genome copies, and viral spreading from infected to bystander cells. These findings were consistent with the results of dec-RVKR-cmk against Chikungunya infection in muscle cells [18]. The effectiveness of dec-RVKR-cmk was observed against both ZIKV and JEV infections at different time points (24, 36, and 48 hpi) in terms of lowering viral titer and viral spreading from infected to neighboring cells. Maximum antiviral activity of dec-RVKR-cmk was perceived at 36 and 48 hpi. In the case of flavivirus, a single round of replication was reported to continue for 16 h [26]. Our study suggests that dec-RVKR-cmk could inhibit virus propagation in a one-step growth cycle, and a mechanism study suggests that it cannot inhibit virus replication, but exerts its inhibitory action on the virus release and next round infectivity. This might be due to inhibition of prM cleavage, which would affect viral packaging or result in accumulation of immature viral particles, while virus release and next round infectivity would be diminished.

Hepatitis C virus (HCV) is also an important member of Flaviviridae but has been placed in a genus separate from the other flaviviruses and it is not clear to what extent HCV envelope proteins behave like those of other members of the family Flaviviridae. As previously reported, proprotein convertases furin was responsible for proteolytic cleavage of pro-TGF-β1 into its bioactive form in HCV-infected cells that positively regulates HCV RNA replication [34]. Based on this study, dec-RVKR-cmk might be active against HCV but with a different mechanism of action.

The phenomenon of redundancy existing between furin and other proprotein convertases to overcome the side effects PCs inhibitor [23,24] suggesting dec-RVKR-cmk could not interfere with the normal cellular function of furin. These findings, together with the observed antiviral activity of dec-RVKR-cmk at a non-cytotoxic concentration in this study, support the possibility of the therapeutic application of the furin inhibitor against flavivirus infection, and our future study of this furin inhibitor will enable verification of therapeutic efficacy in an animal model.

In summary, this is the first study that shows the antiviral activity of dec-RVKR-cmk against flaviviruses (ZIKV and JEV). The observed IC50 and cytotoxicity profile together with time-of-addition and molecular mechanism data suggest dec-RVKR-cmk as a potential candidate for treatment.
